# *Leishmania* promastigotes: building a safe niche within macrophages

**DOI:** 10.3389/fcimb.2012.00121

**Published:** 2012-09-19

**Authors:** Neda Moradin, Albert Descoteaux

**Affiliations:** INRS - Institut Armand-Frappier and Center for Host-Parasite InteractionsLaval, QC, Canada

**Keywords:** *Leishmania*, macrophage, phagosome, virulence, lipophosphoglycan

## Abstract

Upon their internalization by macrophages, *Leishmania* promastigotes inhibit phagolysosome biogenesis. The main factor responsible for this inhibition is the promastigote surface glycolipid lipophosphoglycan (LPG). This glycolipid has a profound impact on the phagosome, causing periphagosomal accumulation of F-actin and disruption of phagosomal lipid microdomains. Functionally, this LPG-mediated inhibition of phagosome maturation is characterized by an impaired assembly of the NADPH oxidase and the exclusion of the vesicular proton-ATPase from phagosomes. In this chapter, we review the current knowledge concerning the nature of the intra-macrophage compartment in which *Leishmania donovani* promastigotes establish infection. We also describe how LPG enables this parasite to remodel the parasitophorous vacuole.

The protozoan *Leishmania* parasitizes phagocytic cells, causing a spectrum of human diseases ranging from a confined cutaneous lesion to a progressive and potentially fatal visceral infection. *Leishmania* is endemic in 98 countries where it constitutes a serious health problem (Alvar et al., 2012). This parasite exists under two distinct developmental stages. Promastigote forms, which develop within sand flies, are inoculated into the mammalian host upon the bloodmeal of the vector. They are internalized by phagocytes where they subsequently differentiate into amastigotes. To do so, promastigotes must avoid being killed by the antimicrobial activities of macrophages. Amastigotes are fully adapted to the conditions encountered within macrophage phagolysosomes and are responsible for the various pathologies associated with the infection. No effective and safe vaccines are available, and current treatment is based on chemotherapy, which is difficult to administer, expensive, and becoming ineffective due to the spread of drug resistance. Understanding the nature and the functional properties of the vacuoles in which both stages of the parasite are internalized and develop is an important step towards the development of novel approaches to prevent and treat leishmaniases.

## The phagolysosome as a replicative niche for amastigotes

Early work by Alexander and Vickerman (1975) and Chang and Dwyer (1976) revealed that *Leishmania* amastigotes multiply in macrophages within compartments that fuse with lysosomes. These seminal discoveries established that in mammals, *Leishmania* resides and proliferates within phagolysosomal compartments of host macrophages. Subsequent work indicated that amastigotes are resistant to the hydrolytic environment prevailing in phagolysosomes (Lewis and Peters, 1977; Chang and Dwyer, 1978). Amastigotes enter macrophages via a Rac1- and Arf6-dependent process, and are found in parasitophorous vacuoles that interact with endosomes and lysosomes and acquire lysosomal features (Chang and Dwyer, 1976; Berman et al., 1979; Antoine et al., 1998; Dermine et al., 2001; Lodge and Descoteaux, 2006). Consistently, vacuoles harboring amastigotes contain numerous lysosomal hydrolases and their membranes are enriched with late endosomal/lysosomal proteins, such as Rab7, LAMP-1, and LAMP-2. The vacuolar H^+^-ATPase present on amastigotes-harboring vacuoles is responsible for the acidic pH (pH 4.7–5.2) (Antoine et al., 1990, 1998; Vinet et al., 2009). In addition, vacuoles harboring amastigotes display molecules characteristic of the endoplasmic reticulum such as calnexin and the membrane fusion regulator Sec22b (Ndjamen et al., 2010). This observation suggests that amastigotes-harboring vacuoles are hybrid compartments composed of both endoplasmic reticulum and endocytic pathway components.

The fact that amastigotes reside in an acidic environment is consistent with their optimal metabolism (respiration, catabolism of energy substrates and incorporation of precursors into macromolecules) at acidic pH (pH 4.0 and 5.5), whereas these activities are optimal at neutral pH for promastigotes (Mukkada et al., 1985). To avoid exposure to oxidants, amastigotes subvert the generation of reactive oxygen species (ROS) within the parasitophorous vacuole through diverse mechanisms including heme degradation and prevention of the NADPH oxidase complex assembly (Pham et al., 2005; Lodge and Descoteaux, 2006). In the latter case, amastigotes evade the phosphorylation of cytosolic p47^*phox*^, a key event for the NADPH oxidase activation during phagocytosis (Lodge and Descoteaux, 2006). Interestingly, *Leishmania donovani* amastigotes disrupt the integrity of lipid microdomains present within the phagosomal membrane, as assessed by the alteration of GM1 distribution and the impairment of flotillin recruitment (Lodge and Descoteaux, 2006). Flotillin is a component of lipid microdomains and is recruited to phagosomes during the maturation process from late endocytic organelles. The mechanisms by which *L. donovani* amastigotes disrupt lipid microdomains and the ensuing consequences on pathogenesis are not known and remain to be investigated. Whereas amastigotes from most *Leishmania* species proliferate in tight individual vacuoles, amastigotes of the *L. mexicana* complex reside in large communal parasitophorous vacuoles (Real et al., 2008). The molecular basis of parasitophorous vacuole enlargement and the consequences for the intracellular survival of these parasites are poorly understood.

## Arrested phagosome maturation by promastigotes

In contrast to amastigotes, promastigotes exist only transiently within mammals. Following their inoculation by sand flies, promastigotes must avoid destruction by the innate immune system of their mammalian hosts in order to differentiate into amastigotes. Hence, promastigotes evade the antimicrobial properties of serum components before being internalized by macrophages. Interestingly, recent studies using an experimental model of natural transmission in mice revealed that a portion of sand fly-transmitted promastigotes may reside transiently within neutrophils before being taken up by dendritic cells and macrophages (Peters and Sacks, 2009). The nature and characteristics of the neutrophil compartments in which promastigotes transit are however unknown.

Serum-opsonized promastigotes enter macrophages predominantly via the complement receptor 3 in a process that mainly depends on the GTPase RhoA (Lodge and Descoteaux, 2005b). Focal exocytosis of host cell membrane originating from endosomes, lysosomes, and the endoplasmic reticulum contributes to the formation of the promastigote-containing phagosomes (Gagnon et al., 2002; Vinet et al., 2009; Forestier et al., 2011). Such supply of membrane from various intracellular compartments may contribute to the formation and the composition of the nascent parasitophorous vacuole.

**Figure 1:**
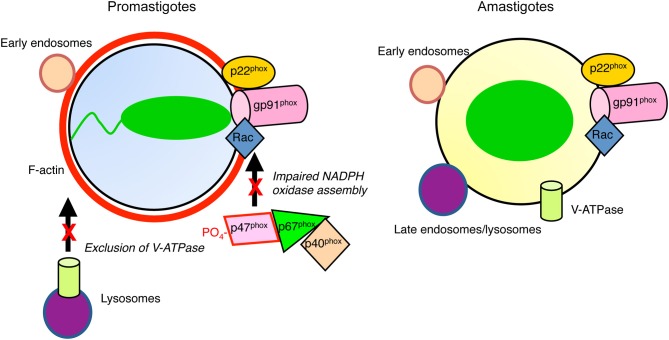
**Diagram illustrating inhibition of phagosome maturation induced by *L. donovani* promastigotes.** Periphagosomal F-actin (red) accumulates, whereas promastigote-harboring phagosomes interact with early endosomes (tan). In contrast to amastigote-containing phagosomes, those harboring promastigotes interact poorly with late endosomes/lysosomes (purple). The V-ATPase is excluded from promastigote-phagosomes, contrasting with those containing amastigotes, which are acidic. Assembly of the NADPH oxidase is impaired in both types of phagosomes.

One mechanism used by promastigotes to evade the microbicidal consequences of phagocytosis is the inhibition of phagolysosome biogenesis (Desjardins and Descoteaux, 1997) (Figure 1). Hence, in contrast to amastigotes, *L. donovani, L. major*, and *L. chagasi* promastigotes are internalized in phagosomes that poorly interact with late endosomes and lysosomes and which display a delayed recruitment of LAMP-1, possibly a consequence of an impaired recruitment of Rab7 (Desjardins and Descoteaux, 1997; Scianimanico et al., 1999; Dermine et al., 2000; Späth et al., 2003; Gaur et al., 2009; Rodriguez et al., 2011). This effect of *Leishmania* promastigotes on phagosome-lysosome fusion is restricted to parasite-containing phagosomes, as the fusion machinery remains operational in infected macrophages (Desjardins and Descoteaux, 1997).

Promastigote-induced phagosome maturation arrest is characterized by a periphagosomal F-actin accumulation (Holm et al., 2001), and by the phagosomal retention of components of the actin polymerization machinery, including Arp2/3, Wiskott-Aldrich Syndrome Protein (WASP), α-actinin, Myosin II, and Nck (Lodge and Descoteaux, 2005a). The Rho-family GTPases, Cdc42, Rac1, and RhoA are also present on promastigotes-containing phagosomes (Lodge and Descoteaux, 2005a; Lerm et al., 2006). The significance of this build up of periphagosomal F-actin is unclear, but it may contribute to the inhibition of phagosome maturation, possibly by interfering with the recruitment of signal transducers and vesicles trafficking to the forming phagolysosome (Lodge and Descoteaux, 2005a; Lerm et al., 2006). However, periphagosomal F-actin accumulation is not observed with all *Leishmania* species. Indeed, F-actin rapidly disassembles from newly formed phagosomes harboring *L. amazonensis* metacyclic promastigotes (Courret et al., 2002). Whether this is related to the fact that *L. amazonensis* induces and resides in spacious communal vacuoles is a possibility that deserves further investigations.

As is the case for amastigotes, promastigotes disrupt lipid microdomains present in the phagosomal membrane (Dermine et al., 2001), as assessed by the inhibition of flotillin recruitment to phagosomes containing promastigotes (Dermine et al., 2001). These membrane microdomains play key roles in various cellular functions, serving as platforms to recruit and concentrate molecules involved in processes such as membrane fusion, generation of microbicidal effectors, and signal transduction (Simons and Vaz, 2004). Targeting and disruption of these structures in phagosomes may thus represent an efficient way for pathogens to subvert the antimicrobial arsenal of macrophages.

## Functional consequences of phagosome maturation arrest by promastigotes

What are the functional consequences of the inhibition of phagosome maturation induced by *Leishmania* promastigotes? An important microbicidal mechanism of macrophages is the generation of ROS (ROS; superoxide anions, hydrogen peroxide) which is mediated by the nicotinamide adenine dinucleotide phosphate (NADPH) oxidase complex. Assembly of the NADPH oxidase complex requires that cytosolic phosphorylated p47^*phox*^ and p40/p67^*phox*^heterodimers associate to form p47/p67/p40^*phox*^heterotrimers prior to their membrane translocation, where they interact with membrane-associated flavocytochrome b558 (Bokoch and Diebold, 2002; Brown et al., 2003). Assembly of this complex at the phagosome membrane allows the generation of high levels of ROS within the phagosome. Disabling the NAPDH oxidase is a virulence strategy used by various pathogens to reduce exposure to oxidants (Vazquez-Torres and Fang, 2001; Allen and McCaffrey, 2007; Harada et al., 2007). In the case of *Leishmania*, uptake of promastigotes triggers the phosphorylation of p47^*phox*^ and the formation of heterocomplexes containing both p47^*phox*^ and p67^*phox*^(Lodge et al., 2006). However, these cytosolic components of the NADPH oxidase complex fail to associate to the promastigote-containing vacuoles. Co-internalization of *L. donovani* promastigotes and IgG-coated erythrocytes revealed that inhibition of ROS production is restricted to promastigote-containing phagosomes. Thus, phagosome maturation arrest induced by *L. donovani* promastigotes enables these parasites to establish infection in an environment devoid of oxidants, which may be favorable to their survival. Interestingly, recruitment of the NADPH oxidase to the phagosome membrane has been shown to limit the proteolytic activity of the phagosome. A tight control of proteolysis within the phagosome is key to an efficient antigen processing and presentation. As activity of the NADPH oxidase plays an important role in the control of antigen presentation (Mantegazza et al., 2008; Savina et al., 2009; Rybicka et al., 2012), its impairment by *L. donovani* promastigotes may contribute to the evasion of the immune system by this parasite.

Phagosome acidification is essential for the acquisition of microbicidal properties and for optimal antigen processing, as most lysosomal hydrolases are optimally active at acidic pH. Acidification is mediated by the vacuolar proton-ATPase (v-ATPase), which is present on various endocytic organelles (Flannagan et al., 2009). The v-ATPase is a multimeric complex consisting in the multi-subunit cytoplasmic V_1_-sector that is responsible for ATP hydrolysis, and of the multi-subunit transmembrane V_0_-sector that pumps protons acrosss the bilayer (Marshansky and Futai, 2008). Several intravacuolar pathogens target this process during establishment of infection. The most notable one is *Mycobacterium tuberculosis*, which secretes a protein tyrosine phosphatase, PtpA, to prevent recruitment of the v-ATPase to the phagosome (Wong et al., 2011). Similar to phagosomes containing *M. tuberculosis*, the v-ATPase is excluded from *L. donovani* promastigote-harboring phagosomes, up to 24 h after phagocytosis (Vinet et al., 2009). This finding provides new insight on our understanding of *Leishmania* biology. In the absence of data on the pH of promastigote-containing phagosomes, it has been assumed that promastigotes initiate infection in an acidic environment and that differentiation of promastigotes into amastigotes is mainly triggered by a rapid exposure to an acidic environment and elevated temperature (Zilberstein and Shapira, 1994; Rosenzweig et al., 2008). Exclusion of the v-ATPase suggests that *L. donovani* promastigotes initiate the differentiation process in a non-acidified environment. Further studies will be required to fully address this issue.

## Promastigote-induced phagosome remodeling requires lipophosphoglycan

An important issue is to understand how *Leishmania* promastigotes can remodel the vacuoles in which they are internalized. Bacterial pathogens such as *Salmonella* and *Legionella* use specialized secretion apparatuses to alter intracellular trafficking and block phagosome maturation (Brumell and Grinstein, 2004; Flannagan et al., 2009). In *Leishmania*, no such secretion systems have been described so far. However, recent work have revealed that *Leishmania* promastigotes release microvesicles into the extracellular milieu to deliver cargo into the infected cells (Silverman and Reiner, 2011; Lambertz et al., 2012). These *Leishmania* exosomes, which contain over 300 proteins, modulate macrophage functions to create an environment permissive for early infection (Silverman et al., 2010; Silverman and Reiner, 2012). The possible involvement of exosomes in promastigote-induced phagosomes remodeling is an attractive hypothesis that remains to be investigated. This being said, so far, the only *Leishmania* molecule known to alter intracellular trafficking and to inhibit phagolysosome biogenesis is the abundant surface glycolipid lipophosphoglycan (LPG) (Desjardins and Descoteaux, 1997).

*Leishmania* synthesizes various glycoconjugates associated to virulence, the most notable belonging to the phosphoglycans family (Descoteaux et al., 1995). These phosphoglycans have in common a unique structure not found in mammals, namely the disaccharide-phosphate Gal(β1,4)Man(α1-PO_4_→6) unit (Descoteaux and Turco, 1999). These unique glycoconjugates can be either secreted (phosphoglycan, proteophosphoglycan, and acid phosphatase), or membrane-bound (lipophosphoglycan, also known as LPG). LPG is the most abundant promastigote surface glyconjugate, with 5 million copies per cell, and forms a dense glycocalyx by covering the entire parasite surface. This molecule is promastigote stage-specific, as it is either strongly down-regulated or absent in the amastigote stage (McConville and Blackwell, 1991; Turco and Sacks, 1991). The abundance, location, and uniqueness of these glycoconjugates are consistent with the functions that LPG plays during the establishment of promastigotes within macrophages.

Structurally, LPG consists of a polymer of the repeating Gal(β1,4)Man(α1-PO_4_→6) unit, linked to a 1-*O*-alkyl-2-*lyso*-phosphatidyl*(myo)*inositol (PI) anchor via a glycan core (Descoteaux and Turco, 1999). At the non-reducing end of the repeating unit moiety is a small cap composed of neutral oligosaccharides, mostly galactose, and mannose residues. The number of repeating units varies from 16 to 30 per LPG molecule, depending on the promastigote developmental stage (procyclic *vs* metacyclic) and species. The PI anchor and the glycan core are extensively conserved among *Leishmania* species (McConville et al., 1990). In contrast, the oligosaccharide cap displays some degree of variability among *Leishmania* species, in both sugar composition and sequence. The most important differences in the structure of LPG among the various *Leishmania* species are found within the repeating units. While *L. donovani* LPG follows the basic Gal(β1,4)Man(α1-PO_4_→6) repeat sequence (Thomas et al., 1992), LPG molecules from other species have additional saccharide side chains branching off the C3 position of the galactose residue.

Using a panoply of *L. donovani* mutants defective in the biosynthesis of LPG, Desjardins and Descoteaux discovered that LPG was the molecule responsible for the inhibition of phagolysosome biogenesis by promastigotes (Desjardins and Descoteaux, 1997). Hence, in contrast to wild type *L. donovani* promastigotes, mutants lacking either LPG or all phosphoglycans were unable alter the fusogenecity of phagosomes toward late endosomes and lysosomes, and did not interfere with the recruitment of the late endocytic and lysosomal markers Rab7 (Scianimanico et al., 1999). These findings are consistent with the observation that amastigotes, which do not make LPG, are internalized into compartments that acquire lysosomal features (Dermine et al., 2000; Courret et al., 2002), including the v-ATPase (Vinet et al., 2009).

Further studies revealed the importance of the length of the LPG repeating unit moiety. Hence, a minimal length appears essential to perturb membrane properties, as was shown in a viral syncytia formation assay and in a PKC membrane association assay (Easterbrook et al., 1995; Giorgione et al., 1996). Consistently, a *L. donovani* mutant expressing truncated forms of LPG with only 3–5 repeating units was unable to inhibit phagosome-endosome fusion (Desjardins and Descoteaux, 1997). In contrast, the level of structural complexity of this moiety, such as the presence of oligosaccharide side-chains, does not affect membrane properties. Indeed, a *L. major* mutant defective in LPG oligosaccharide side-chain biosynthesis was able to impair phagosome-endosome fusion to the same extent as wild-type *L. major* promastigotes (Dermine et al., 2000).

Clearly, LPG exerts a profound influence on the composition and properties of the promastigote-harboring phagosomes. From a cell biology stand point, in addition to understanding the biology of *Leishmania* parasites, this discovery provided a novel and unique system to investigate the process of phagolysosome biogenesis. For both reasons, one important issue was to elucidate the mechanism(s) by which a single microbial-derived glycolipid can affect so profoundly phagosome maturation.

## The impact of lipophosphoglycan on phagosome properties

Periphagosomal accumulation of F-actin induced by LPG (Holm et al., 2001) is the consequence of an abnormal retention of the Rho-family GTPase Cdc42 at the phagosome. This role for Cdc42 was revealed by expressing the dominant-negative Cdc42N17 mutant in RAW 264.7 macrophages, which inhibited LPG-mediated periphagosomal F-actin accumulation (Lodge and Descoteaux, 2005a; Lerm et al., 2006). Interestingly, the host cell machinery involved in actin polymerization and cytoskeleton rearrangement, such as WASP, Arp2/3, Nck, α-actinin, and myosin II, are retained at the phagosome in a LPG-dependent manner, and this can be reversed by the Cdc42N17 mutant (Lodge and Descoteaux, 2005a). LPG also interferes with the recruitment of Protein Kinase C (PKC)-α to the phagosome membrane (Holm et al., 2001). PKC-α was shown to participate in periphagosomal F-actin breakdown (Holm et al., 2003) and in the regulation of phagosome maturation (Allen and Aderem, 1995; Aderem and Underhill, 1999; Ng Yan Hing et al., 2004). Whether LPG-mediated exclusion of PKC-α from *L. donovani* promastigote-containing phagosomes contributes to the periphagosomal accumulation of F-actin remains to be demonstrated. Similarly, the consequences of periphagosomal F-actin accumulation on phagosome functions remain to be investigated.

Another mechanism by which LPG exerts its action on phagosome maturation implicates the transfer of LPG from the parasite surface to lipid microdomains present in the phagosome membrane (Tolson et al., 1990). This causes a disorganization of these structures and prevents the formation of new lipid microdomains after phagocytosis. Phagosomal lipid microdomains are central to the recruitment/assembly of the NADPH oxidase and the v-ATPase, and are involved in the regulation of phagosome-endosome fusions (Dermine et al., 2001; Shao et al., 2003; Vilhardt and Van Deurs, 2004). How lipid microdomains regulate interactions between phagosomes and the endocytic system is unclear. However, the observations that proteins involved in membrane fusion are located in lipid microdomains are consistent with these structures acting as fusion sites (Gil et al., 2005; Kay et al., 2006). Insertion of LPG into lipid microdomains via its GPI anchor allows the negatively charged Gal(β1,4)Man (α1-PO_4_→6) polymer to directly interfere with the clusterization of molecules into these microdomains (Dermine et al., 2005; Winberg et al., 2009). One direct consequence of LPG-mediated microdomain disorganization is the exclusion of the membrane fusion regulator Synaptotagmin (Syt) V from the phagosome (Vinet et al., 2009). Syt V plays a regulatory role in phagocytosis (Vinet et al., 2008), as well as in phagosome maturation, by controlling the acquisition of the v-ATPase and of cathepsin D (Vinet et al., 2009). Thus, exclusion of Syt V from the phagosome membrane by LPG abrogates recruitment of the v-ATPase and impedes phagosome acidification (Vinet et al., 2009). Targeting the phagosome fusion machinery thus represents an efficient way to create an intracellular niche favorable to the establishment of a pathogen.

Shedding or secretion of glycans as a virulence mechanism to modulate phagosome maturation has been described for other intracellular pathogens, including *M. tuberculosis, Brucella abortus, and Legionella pneumophila*. Similar to LPG, the cyclic β-1,2-glucans of *B. abortus* and the lipoarabinomannan of *M. tuberculosis* impair phagolysosomal biogenesis by disrupting host cell lipid microdomains (Arellano-Reynoso et al., 2005; Welin et al., 2008). In the case of *L. pneumophila*, transmissive forms shed LPS-containing membrane vesicles that inhibit phagosome fusion with degradative lysosomes (Fernandez-Moreira et al., 2006). The exact mode of action is not known. Whether these various bacterial glycans act by disabling the phagosome membrane fusion machinery remains to be further explored.

## Concluding remark

Similar to other intracellular pathogens, *Leishmania* promastigotes block the phagosome maturation process and create an environment which may be propitious to promastigote-to-amastigote differentiation. The surface glycolipid LPG plays a central role in this process. Further studies will be required to determine whether other *Leishmania* molecules are involved in the phagosome remodeling induced by promastigotes. Defining the functional properties of the promastigote-harboring vacuoles may also provide new insights into our understanding of the biology of *Leishmania* parasites, as well as the biology of phagolysosome biogenesis.

### Conflict of interest statement

The authors declare that the research was conducted in the absence of any commercial or financial relationships that could be construed as a potential conflict of interest.
